# ‘My coupons are like gold’: experiences and perceived outcomes of low-income adults participating in the British Columbia Farmers’ Market Nutrition Coupon Program

**DOI:** 10.1017/S1368980021001567

**Published:** 2022-02

**Authors:** Stéphanie Caron-Roy, Sayeeda Amber Sayed, Katrina Milaney, Bonnie Lashewicz, Sharlette Dunn, Heather O’Hara, Peter Leblanc, Bonnie Fournier, Kim D Raine, Charlene Elliott, Rachel JL Prowse, Dana Lee Olstad

**Affiliations:** 1 Faculty of Kinesiology, University of Calgary, Calgary, AB, Canada; 2 Department of Community Health Sciences, Cumming School of Medicine, Research and Wellness Building, 3280 Hospital Drive NW, University of Calgary, Calgary, AB T2N 4Z6, Canada; 3 British Columbia Association of Farmers’ Markets, Vancouver, BC, Canada; 4 School of Nursing, Thompson Rivers University, Kamloops, BC, Canada; 5 School of Public Health, University of Alberta, Edmonton, AB, Canada; 6 Department of Communication, Media and Film, University of Calgary, Calgary, AB, Canada; 7 Chronic Disease and Injury Prevention, Public Health Ontario, Toronto, ON, Canada; 8 Dalla Lana School of Public Health, University of Toronto, Toronto, ON, Canada

**Keywords:** Farmers’ market, Food subsidies, Food insecurity, Qualitative research, Diet quality, Adults

## Abstract

**Objective:**

The British Columbia Farmers’ Market Nutrition Coupon Program (FMNCP) provides low-income households with coupons valued at $21/week for 16 weeks to purchase healthy foods in farmers’ markets. Our objective was to explore FMNCP participants’ experiences of accessing nutritious foods, and perceived programme outcomes.

**Design:**

The current study used qualitative description methodology. Semi-structured interviews were conducted with FMNCP participants during the 2019 farmers' market season. Directed content analysis was used to analyse the data, whereby the five domains of Freedman *et al.*’s framework of nutritious food access provided the basis for an initial coding scheme. Data that did not fit within the framework’s domains were coded inductively.

**Setting:**

One urban and two rural communities in British Columbia, Canada.

**Participants:**

Twenty-eight adults who were participating in the FMNCP.

**Results:**

Three themes emerged: autonomy and dignity, social connections and community building, and environmental and programmatic constraints. Firstly, the programme promoted a sense of autonomy and dignity through financial support, increased access to high-quality produce, food-related education and skill development and mitigating stigma and shame. Secondly, shopping in farmers' markets increased social connections and fostered a sense of community. Finally, participants experienced limited food variety in rural farmers' markets, lack of transportation and challenges with redeeming coupons.

**Conclusions:**

Participation in the FMNCP facilitated access to nutritious foods and enhanced participants’ diet quality, well-being and health. Strategies such as increasing the amount and duration of subsidies and expanding programmes may help improve participants’ experiences and outcomes of farmers' market food subsidy programmes.

In 2017–2018, food insecurity, defined as the inadequate or unreliable access to food due to financial constraints, was experienced by over 12 % of households in Canada^(1)^. This problem exists in every Canadian province, including British Columbia (BC), where one in eight households experienced some level of food insecurity in 2017–2018^(1–4)^. Experiences of food insecurity can range from worrying about running out of food, to compromising diet quality, to missing entire meals or not eating for an entire day^(5,6)^. As inadequate income is the most important determinant of food insecurity^(6)^, individuals with lower incomes have higher rates of food insecurity than those with higher incomes^(5,7–9)^.

Evidence has shown that food insecurity and poor diet quality are interrelated. Individuals living in food insecure households tend to consume fewer nutrient-rich foods^(10–15)^ and have poorer mental, social and physical health^(9,16–26)^. Current evidence suggests that targeted policies and interventions that seek to improve access to nutritious foods for low-income households may help to reduce food insecurity and improve diet quality. This may lead to better overall health and corresponding reductions in avoidable healthcare expenditures^(27–29)^.

One way to increase access to nutritious foods is through the provision of food subsidies for low-income, food insecure households. Evidence suggests that subsidies to purchase healthy foods in supermarkets can increase fruit and vegetable intake^(30,31)^. Similar benefits may be realised through subsidies to purchase fruits and vegetables in farmers’ markets (FMs)^(32–40)^. For example, Herman *et al.* ^(41)^ found that women enrolled in a federally funded FM food subsidy programme in the USA that provided $10/week to purchase fresh produce in FMs consumed more fruits and vegetables than those in a control group who received coupons of lesser value for non-food-related products. FMs are also unique social spaces that can foster a sense of community and thereby help to improve social and mental well-being^(36,42–46)^.

Participants’ experiences in these programmes have been explored in qualitative studies. For instance, studies identified several barriers for low-income participants to shop and utilise subsidies in FMs, including limited access to transportation, work schedules that conflict with market hours of operation and the perception that FMs were more expensive than other retailers^(47–50)^. Ritter *et al.* ^(51)^ also reported that voucher systems might set participants apart from the general population, thereby creating stigma. On the other hand, positive experiences with FM subsidy programmes were identified, including greater exposure to fruits and vegetables and increased financial resources^(47–49,51)^.

Qualitative studies have also explored participants’ perceived outcomes of FM food subsidy programmes. Participants reported outcomes such as greater fruit and vegetable intake, increased likelihood of trying new foods, educational opportunities for children and perceived improvements in their quality of life^(47,48,52)^. Savoie-Roskos *et al.* ^(48)^ also found that participants emphasised the value of FMs for building social connections between farmers and community members. Conversely, evidence indicates FM subsidy programmes may also have unintended negative outcomes such as increased financial stress among participants when programmes end^(53)^.

Despite growing interest and ongoing support of FM food subsidy programmes across North America, there is limited knowledge on factors influencing participants’ experiences, access to, use and outcomes of these programmes. Including the voices of low-income participants is essential to improving FM food subsidy programmes to better meet their needs^(54)^. Current evidence suggests positive dietary and economic benefits for participants; however, psychosocial outcomes such as social connections, community connectedness and mental well-being have not been adequately explored. Lastly, it is worth noting that all existing qualitative studies have been conducted in the USA, and the transferability of these findings to other nations is unclear due to differences in policies and programmes for low-income households, and discrepancies in FM accessibility and affordability between nations.

## Programme overview and objectives

The BC Farmers’ Market Nutrition Coupon Program (FMNCP) is the only government funded FM food subsidy programme in Canada^(55)^. It is overseen by the BC Association of Farmers’ Markets, with support from the BC Ministry of Health and the Provincial Health Services Authority. The programme provides participants with 16 weeks’ worth of coupons valued at $21/week to purchase fruits, vegetables, dairy, meats, fish, eggs, nuts and fresh herbs in participating BC FMs from June to November^(55)^. As most previous qualitative studies of FM food subsidy programmes in the USA have investigated fruit and vegetable subsidies exclusively^(47,49–52)^, the current study is unique by examining experiences and outcomes associated with subsidies that can be used to purchase a variety of healthy foods in FMs. The FMNCP also includes nutrition skill-building activities (e.g., pre-natal nutrition programmes, cooking classes). FMNCP coupons are offered in $3 increments which are deemed flexible enough for small purchases and allow for more effective distribution and handling than $1 coupons.

The FMNCP is delivered through a collaboration between local FMs and community partner organisations which provide public health-related services in a specific community. Within the FMNCP, community partner organisations are responsible for enrolling low-income households into the programme using community-specific thresholds, and distributing coupons. Community partner organisations are expected to manage programme enrollment based on community needs. For instance, those working with pregnant women will enroll new participants into the programme every year, while others may enroll the same participants year over year (e.g., those who work with seniors). Others draw participants from a pool of applications from the community every year and create waitlists based on demand. Community partner organisations are also asked to provide nutrition skill-building activities to FMNCP participants; however, participation is not mandatory^(55)^. In 2019, the FMNCP served 5404 households, including low-income families, pregnant women and older adults in seventy-eight urban and rural communities across BC^(56)^.

This qualitative study was co-designed with the BC Association of Farmers’ Markets to: (1) describe participants’ experiences of accessing nutritious foods, including facilitators and barriers, and (2) explore participants’ perceived programme outcomes and how these outcomes were achieved.

## Methods

### Methodology and theoretical framework

Qualitative description methodology was used. As a methodological approach, qualitative description aims to provide rich descriptions of participants’ accounts and attempts to interpret findings without moving too far from literal descriptions^(57–59)^. Data generation and analysis were guided by Freedman *et al.*’s^(60)^ theoretical framework of nutritious food access. This framework draws attention to the variety of interrelated factors that influence access to nutritious foods for low-income households. The model includes five domains: (1) economic, (2) spatial–temporal, (3) service delivery, (4) social and (5) personal factors. The framework informed development of interview questions and a preliminary coding scheme.

### Participant recruitment and data generation

Participant recruitment occurred with the collaboration of five volunteer community partner organisations from one urban and two rural (areas with ≤10 000 people^(61)^) communities. Participants were purposively recruited by community partner organisations via face-to-face discussions, emails and social media platforms, with the goal of recruiting a total of 25–30 participants. Based on sample sizes reported in qualitative descriptive studies^(62)^ and previous qualitative studies of FM food subsidy programmes^(47–52)^, we anticipated that we would reach data saturation after twenty interviews. To be eligible, individuals had to be ≥ 18 years of age, from low-income households participating in the FMNCP during the 2019 FM season (June–November) and the primary food shopper for their household. As $21/week may not adequately cover the needs of larger households, eligible participants were required to have eight or less people living in the household. Participant recruitment occurred until no new concepts were being identified in the data.

Prior to conducting interviews, the interview guide was pre-tested with two FMNCP participants over the phone and questions were subsequently modified. As both interviews captured the essence of participants’ experiences and perceived outcomes, these data were included in the analysis. Two researchers (S.C.-R. and S.A.S.) then conducted in-person, semi-structured interviews with twenty-six participants from August to September 2019. Interviews lasted 45–60 min. Sample interview questions and probes are presented in Table 1. Following each interview, participants were asked to provide demographic information such as sex, age, ethnicity, household composition, education, employment status and annual household income. Participants also reported food insecurity using a two-item screener^(63)^. Participants were offered a $25 cash incentive for participating in the study.

**Table 1:** Interview questions and probes for semi-structured interviews with twenty-eight adults participating in the 2019 British Columbia Farmers’ Market Nutrition Coupon Program

Areas of interest	Interview questions	Sample probes
Programme experiences	What have been your experiences with the FM nutrition coupon programme so far?	What makes it easy for you to participate in the programme? What makes it difficult or challenging to participate in the programme?
Shopping in FMs	Why do you visit FMs?	What do you do when you visit a FM? What do/don’t you like about shopping in FMs? Why? Probes queried each of the five domains in Freedman *et al.*’s^(60)^ framework (e.g., interactions with farmers, vendors and other FM customers, transportation, perceived food costs, etc.)
Skill-building activities	Are you aware of the nutrition skill-building activities with your community partner organisation?	What types of activities have you taken part in? What do you think of the nutrition skill-building activities you have attended so far?
Perceived programme outcomes	How has the programme (i.e., coupons and skill-building) affected you?	How has the programme affected your social life/mental health/finances/eating/the people who live with you? Has the programme affected you in any negative ways? If so, can you explain?

FM, farmers’ market.

### Data analysis and rigour

All interviews were recorded and transcribed verbatim with assistance of an artificial intelligence (AI) transcription app^(64)^. Analysis began with repeated listening of audio recordings to become familiar with the data. S.C.-R. corrected discrepancies in the AI transcription app and entered transcripts in NVivo (version 12.6, University of Calgary) to manage and organise coding during data analysis. Pseudonyms were assigned to each participant to protect anonymity and confidentiality.

Directed content analysis^(65)^ was used to analyse the data, whereby Freedman *et al.*’s^(60)^ framework provided the basis for an initial coding scheme. Data that did not fit within the framework’s domains were coded inductively. S.C.-R. and S.A.S. coded the first three interviews independently and subsequently met to reach consensus on a coding scheme. Researchers followed coding and interrater reliability procedures outlined by Jackson *et al.* ^(66)^ and Hruschka *et al.* ^(67)^. This involved using coding comparisons to identify codes with low agreement between researchers, which were then reviewed to enhance coding practices. Both researchers independently analysed another three interviews and met following each analysis to discuss discrepancies and finalise the coding scheme. Once all remaining transcripts were coded, S.C.-R. collated and categorised codes to generate themes and subthemes, which were then presented to the research team to assist in defining and naming themes.

Several strategies to enhance rigour were employed. The involvement of two researchers in the data generation and analysis process enhanced trustworthiness of findings through peer debriefing and investigator triangulation^(68–71)^. In addition, an audit trail was maintained, including field notes and researcher reflections, to provide a transparent description of study processes^(72,73)^. Finally, the use of thick verbatim extracts from participant interviews helped to ensure findings remained true to participants’ accounts^(74)^.

## Results

Participant characteristics are presented in Table 2. Three themes, with related subthemes, describing participants’ programme experiences and their perceived outcomes of the programme were generated from the data analysis process: (1) autonomy and dignity, (2) social connections and community building and (3) environmental and programmatic constraints. These results focussed mainly on convergent findings. A few divergent findings emerged from the data, which are noted throughout.

**Table 2:** Demographic information obtained during semi-structured interviews with twenty-eight adults participating in the 2019 British Columbia Farmers’ Market Nutrition Coupon Program

Characteristics	Number (*n*)	Percentage (%)
Sex		
Female	22	79
Male	6	21
Age (years)		
19–59	19	68
≥60	9	32
Household composition		
1 person	10	36
2–4 persons	12	43
5–8 persons	6	21
Number of children living in the home (<19 years)		
None	13	47
1–3	11	39
4–5	4	14
Ethnicity		
White	12	43
First nations	8	28
Métis	3	11
Other	5	18
Education		
< High school diploma	3	11
High school diploma	10	36
Trade, college or non-university degree	7	25
University below bachelor’s degree	2	7
Bachelor’s degree	3	11
> Bachelor’s degree	2	7
Undisclosed	1	4
Employment status		
Full-time, part-time or self-employed	8	29
Retired	7	25
Unable to work	6	21
Unemployed and not looking for work	5	18
Homemaker	2	7
Annual household income (CAD $)		
1–9999	2	7
10 000–19 999	11	39
20 000–29 999	8	28
30 000–39 999	1	4
40 000–49 999	4	14
50 000–59 999	1	4
60 000–69 999	1	4
Food insecure*		
Yes	19	68
No	9	32

* Based on Hager *et al.*’s^(63)^, two-item screener to identify households at risk of food insecurity.

### Theme 1. Autonomy and dignity

#### Financial support

Participants reported the financial support provided by the FMNCP supplemented their food budgets, which allowed them to purchase more fresh produce. For instance, Ava (single-adult household, age 30) reported how she was able to purchase more food with the coupons: ‘It’s really hard to figure out what to budget for, after rent, food is my biggest part of my budget. Otherwise, like I don’t have much money in the bank… the coupon program really helps, and I have more food in my fridge. My fridge is actually full’.

The programme also enhanced participants’ sense of financial autonomy as they could often divert funds towards other living expenses. Although some purchases were for items like clothes for themselves and their children, Claire (lone-parent household, one child, age 44), reported that she allocated funds that she normally would have spent on food towards extra-curricular activities for her daughter: ‘With the coupons, being able to buy the vegetables and stuff like that, it also gives me that little extra money throughout the month to be able to send [my daughter] swimming. I still can’t afford to take her and put her into dance classes or anything like that. But at least she can go swimming a couple of times a month or, you know, maybe go to the movies’.

Participants often described the programme as a ‘godsend’, ‘a miracle’ or ‘like gold’, which highlighted the exceptional perceived worth of the coupons and reaffirmed their considerable financial impact for many participants.

#### Food and diet quality

Participants reported greater access to fresh, local produce and protein-rich foods, including fruits, vegetables, eggs and meats. The programme also allowed participants to try new foods and purchase a greater variety of foods. Claire (lone-parent household, one child, age 44) described how the programme helped improve the quality of her diet: ‘Now I’m more apt to have more vegetables and stuff in my house, where if you go to the grocery store, it’s so expensive there and I’m on disability… It’s mostly processed foods when I have to shop at the store. With the coupons, at least I now have a better chance of having the healthy food’. Jeanette (single-adult household, age 67) was eating a mostly vegan diet due to financial restrictions, and the coupons helped reintroduce protein-rich foods into her diet: ‘So I started eating [meat and eggs] because I had the coupons… I added eggs back in right away. And I started even buying the homemade sausages from these people’.

Participants commonly expressed a preference for foods purchased in FMs; the quality and freshness were often perceived as superior to foods available in grocery stores and/or food banks. Janice (single-adult household, age 56), for example, noted a stark contrast in the quality and shelf-life of foods sold in FMs *v*. foods obtained at the food bank: ‘I eat better foods, and hardly anything goes bad. Like you know how you have stuff in your fridge, if you don’t pay attention, you can be throwing stuff out… when I get stuff from the food bank, it goes bad really fast. And this stuff here does not go bad really fast’. Foods sold in FMs were also perceived as healthier, more flavourful and pesticide-free. According to Mark (two-parent household, one child, age 24): ‘[The food at the FM] makes me feel a lot healthier. And the stuff that you can get at the FM is really high in nutrients. It’s not sourced far away, it’s not sitting on shelves for a long time, and they’re all local guys that do their own growing. So it’s really nutritious and it’s good’. One contradictory account emerged, in which a participant perceived that foods sold in grocery stores were higher quality than foods sold in their local FM.

The ability to access high-quality and local foods at FMs that participants could not otherwise afford brought a sense of pride and dignity. This was conveyed by Samira (two-parent household, three children, age 36): ‘[There is] something empowering about getting the best ingredient to feed your family. So whenever I’m able to shop, and I know that I have the freshest product, I have the best in my bag. I just feel, you know, proud’.

#### Food and nutrition knowledge and skill development

Shopping in FMs offered many opportunities for nutrition education and skill development. For instance, Chesa (lone-parent household, one child, age 33) shared her experiences of interacting with farmers and vendors: ‘When you go there in a FM, they will explain it to you how to cook it… While in the [grocery stores], no… If you ask them because they’re very busy, just grab the vegetables, and the fruits. But [in the FMs], if you ask them, they will explain it to you’. Chesa’s experiences were echoed by others, suggesting these types of interactions were not only common in FMs but also distinct from experiences in grocery stores.

Participants with children (*n* 15) often involved their children in shopping and preparing foods purchased in FMs. For Genesis (lone-parent household, four children, age 30), these experiences ‘made the children more interested in the actual ingredients’ and assisted in introducing new foods to her eldest daughter who is a picky eater: ‘Actually seeing, and picking, and holding the ingredients has made her more interested in eating the ingredients at home… So she’s actually trying more foods now… It’s made me more aware of how important touching and feeling the food is, and seeing where it comes from’. In addition, participants reported that farmers and vendors played a key role in educating their children about different foods, and how to grow and prepare them.

A few divergent experiences were reported, as not all participants gained knowledge or skills from shopping in FMs. Participants, like Luis (single-adult household, age 73), discussed how his prior food and nutrition knowledge helped him make healthy food choices: ‘I’ve just eaten what I’ve eaten all my life, you know. I don’t have to go to a program to find out what’s good because all the stuff that I have eaten all my life has kept me going this far, kept me as healthy as I am’.

#### Mitigating stigma and shame

Some participants (*n* 9) described experiences of stigma and shame. Experiences of stigma reported by participants were often internalised and associated with receiving financial support from a programme. Others anticipated negative views or actions from farmers, vendors and FM patrons when initially redeeming coupons in FMs. This anticipation tended to cause feelings of embarrassment among participants. Finally, a few noted experiences of stigma which were enacted by farmers, vendors and FM customers. These included being looked at or noticed when redeeming FMNCP coupons in FMs, being questioned about the coupons by other FM shoppers and not feeling welcomed to purchase products at one FM booth.

Despite some stigmatising experiences, there were several factors that protected most participants from experiencing stigma and shame when redeeming coupons in FMs. First, these feelings were often minimised through interactions with farmers and vendors. According to participants, farmers and vendors were ‘cheerful’ when receiving coupons, and ‘treat it like cash’, as coupons represented a source of income for them. Keith (two-parent household, four children, age 41) discussed his experiences: ‘I wouldn’t say anyone has made me feel inferior or less because I’m accessing a program, I think that’s something I might have projected on myself. But everyone that receives them are very pleased. Very, very happy. Because as far as my understanding, it’s as good as money to them anyways’. Secondly, Chris (two-parent household, five children, age 43) explained how the large size of some communities helped to minimise potential stigma associated with coupon use: ‘You go to the FM in [urban community], a hundred thousand people, everybody’s using them… There is no stigma around it. It’s just as probably more accepted than cash just because the vendors know, if I got this piece of paper, that’s as good as gold’. Chris’ statement also highlighted the perceived value attributed to coupons by farmers and vendors, which may have acted as additional factors that protected participants from experiencing feelings of stigma and shame when redeeming their coupons. Finally, participants felt a sense of community when shopping in FMs. For Ava (single-adult household, age 30), this mitigated stigma attached to being low-income: ‘It’s really appreciated because sometimes, if you’re a low-income, you feel like you’re less than, you’re like scum sometimes, like just getting by but when you go to the market with coupons, you feel like you’re part of the community. You don’t feel like low-income’.

### Theme 2. Social connections and community building

#### Social environment

Many participants perceived FMs as unique social environments that fostered connections within and outside the household. Mark (two-parent household, one child, age 24) described how shopping in FMs became an outing for his family and an opportunity to meet new people: ‘It’s really good. I mean, it gets us out on a Saturday and normally, we’re pretty secluded, we don’t tend to do too much social interaction. So just to get out and be a part of that culture is really impactful for us, and for our family… They’re all just really nice people that you meet at the FM. So it helps us to create more bonds’. These types of experiences were common amongst adults with children; FMs provided opportunities for families to spend time together. Genesis (lone-parent household, four children, age 30) often referred to going to the FM with her children as ‘a big adventure’. Similarly, for single, older adults (*n* 8), social interactions in FMs were frequent. As a new member of her community, Jeanette (single-adult household, age 67), perceived the FM as a place to meet new people in her area: ‘There’s a certain social aspect of it now too, because now I’ve gotten to know the vendors and they recognize me now as a regular customer. And I’m starting to actually meet people and make connections as well, which is important when you’re a newcomer to the area’. A few participants expressed divergent accounts and did not perceive FMs as having unique social environments. This was the case for participants that did not shop in FMs on a regular basis and did not interact closely with farmers, vendors and other FM patrons when shopping in FMs.

As alluded to earlier by Mark, FMs have a distinct culture that may foster social bonds within communities. FMs were occasionally described as ‘festive’, or as Mary (single-adult household, age 62) explained, ‘it’s like going to the carnival or something’. According to Keith (two-parent household, four children, age 42), FMs attracted customers looking for similar shopping and social experiences, thereby influencing the overall culture and environment: ‘The environment is busy. It is energetic, yet calm. People don’t rush through the crowd, they move quite fluidly. You don’t see grumpy people there… you go to the grocery store, you’ll see grumpy people. When you go to the market, it’s different. People are there for an experience and local support, and it reflects in everybody’s behaviour in that area’.

Shopping in FMs also had positive social and mental health outcomes for participants. Several noted improvements in their social and mental well-being as a result of going out and interacting with others. These benefits were also perceived by participants struggling with anxiety, social isolation and/or depression (*n* 8). For instance, Martha (single-adult household, age 40), who struggled with depression, explained the benefits of being around crowds: ‘My counsellor kept saying she’s like “go out in public and do something"… [The FM] was my go-to, you know, reintroducing myself to the public and being in crowds with people I didn’t know’. Moreover, participants like Gary (two-parent household, two children, age 49), who struggled with social anxiety, became more comfortable around crowds: ‘Now I’m out there being sociable, and I’m kind of out of my comfort zone more, and then I get out a little bit more, and more every time I go there’.

#### Sense of community and reciprocity

The FMNCP enhanced participants’ sense of belonging and connectedness to their community and expanded their social networks. Samira (two-parent household, three children, age 36) explained: ‘[I like] the interaction with the farmers. You get to know people in the community and you get to know what’s going on, like sometimes you hear about an event, and things like that… people tell you about their travelings and you make friends’.

This sense of community was also present for participants who identified as First Nation or Métis (*n* 11), including Lillian (lone-parent household, four children, age 33), who felt that interacting with farmers and vendors allowed her to learn more about traditional Indigenous foods: ‘You could sit there and make a friend right away. Like there’s a connection right away because you want to learn, like you need to be educated about different vegetables or fruit… My mom passed away a long time ago so I didn’t get to really experience preserving food with her. Just fish, like salmon. So just asking questions and stuff, I felt like, you know, connected to a community that will always be there’. This learning and sense of community was further enhanced by attendance to nutrition skill-building activities offered by community partner organisations where participants like Sarah (lone-parent household, one child, age 31) attended workshops about traditional Indigenous food practices and socialised with other participants and their children: ‘I like to come to on Wednesdays because there’s usually a lot of other babies here. So I want [son] to socialize with them. And then I just sign up for other programs that interest me. Like I’m canning tomatoes on Friday’.

Finally, the ability to support local farmers cultivated feelings of reciprocity or ‘returning the favour’ among participants who knew that they were re-investing provincial funds into their local economy. Isabelle (single-adult household, age 68) described the benefits of the coupon programme to participants and local producers: ‘It’s knowing that the local farmers are benefiting as well. I’m not the only one that’s benefiting, you know, farmers and producers, that sort of thing. So it’s enhancing their life and enhancing mine’.

#### Social connections beyond farmers’ markets

Opportunities for social interactions extended beyond FMs. As a result of the financial support of the FMNCP, Esthel (single-adult household, age 63) was able to share foods more frequently with her friends: ‘I’m also able, because of the coupon program to share my food more… so I can cook a meal, and invite friends over now, because I have enough food to share. So even though my social life at the actual market hasn’t changed, it’s changed at home because I can have friends over more often or attend a potluck without grief’. In addition, some participants shared some of their coupons with their friends or others in the community.

### Theme 3. Environmental and programmatic constraints

#### Food variety and transportation

The size and variety of foods available in FMs varied by location. According to participants living in a rural community with a small, local FM (*n* 10), the FM had very few food booths, limiting the variety of foods available for purchase with the coupons. Sarah (lone-parent household, one child, age 31) explained: ‘I feel like there’s not much selection at the FM. There’s tomatoes, cucumbers, some squash, and then like the peaches. So there’s not a lot of selection there, really’.

Due to the lack of variety in this local FM, participants often reported the desire to travel to larger, urban FMs to access a greater variety of foods. Although participants with yearly household incomes over $40 000 (*n* 6), such as Robin (two-parent household, two children, age 38), could afford to travel to the nearest urban centre, transportation was an issue for others: ‘Like really with [rural community], and just with what it provides, it’s just limited and so that was really tough. And unless you have the ability to travel, or you know people that do, then you have access to more stuff. But not everybody does’.

#### Programme limitations

A few limitations concerning programme access, nutrition skill-building activities and coupon logistics were discussed. First, participants like Gary (two-parent household, two children, age 49), reported waiting years to access the programme due to long waitlists: ‘My sister told me about it like a couple years ago, but I tried to get on, I was like 24th in line. Then the following year I was like 12th in line. And this year I was 6th in line and I was glad I made it’. Second, not all community partner organisations offered nutrition skill-building activities to participants. Three out of five community partner organisations in our study offered sessions such as cooking lessons, pre- and post-natal nutrition classes. As a result, a few participants (*n* 6) did not have access to any activities, while others (*n* 12) were either unaware that sessions were offered or were unable to attend due to conflicting schedules.

Some participants experienced challenges when redeeming coupons because they were provided in $3 increments. Since farmers and vendors were prohibited from giving change from purchases made with coupons, participants like Chesa (lone-parent household, one child, age 33) were often required to make purchases in $3 increments unless they were able to supplement with their own money: ‘I only have $6, and the amount of the vegetable is $7. And then we can give them another $3 [coupon], and they don’t have change… So I hope there’s a denomination like $1 so that we can add up $7, instead of $9’. Therefore, many participants recommended creating $1 and $2 coupons to provide greater purchasing flexibility.

A few participants identified difficulties accessing certain staple foods with the coupons. For instance, Esthel (single-adult household, age 63) suggested adding bread as an option for purchase with the coupons as she consumed it on a regular basis: ‘Maybe some breads would be nice to have. Even if it’s not like goodies, and cookies, and things I can see maybe that wouldn’t be part of the program. But bread, yeah… because we eat bread daily’. Others also suggested adding honey, jams, preserves, baked goods and prepared foods.

Finally, a few participants suggested increasing the amount and duration of food subsidies. Participants with larger families (i.e., > 4 members in the household) recommended increasing the weekly subsidy to better meet their needs. For instance, Chris (two-parent household, five children, age 43) suggested increasing the number of coupons available for meat products: ‘They give you, I think, three of the coupons, or is it only two, that you can use for purchasing meat. Which, just for my family, maybe 50 % might be better’. A few participants (*n* 4) also suggested extending the programme beyond 16 weeks. René (two-adult household, age 60) explained: ‘It would be nice if [the FMNCP] was longer, it would be nice if you could start in March and then go through to October. You know, that would be a nice, long period of time to provide coupons for people’.

Although programme limitations were highlighted by participants, other participants found the programme easy and simple to use and did not suggest any changes. This was noted by Flip (single-adult household, age 65): ‘I’m pretty impressed… I haven’t thought of anything that needs to be changed. I go in, tell them my name, I sign a little thing, and I get my coupons. It’s so simple’.

## Discussion

The current study aimed to explore participants’ experiences and perceived programme outcomes of the FMNCP. Our findings illustrate factors that facilitated and constrained access to healthy foods and highlight the programme’s positive influence on participants’ diet quality, well-being and health.

Findings suggest that the FMNCP enhanced food access by addressing several domains of Freedman *et al.*’s^(60)^ framework, including primarily economic and service delivery factors. For instance, participants had greater financial access to fruits, vegetables and protein-rich foods, and perceived foods purchased in FMs as higher quality than those available in other food venues. These findings are consistent with qualitative studies of FM food subsidy programmes in the USA in which participants reported increased fruit and vegetable intake, and greater access to fresh, high-quality produce^(48–50)^. In addition, our results draw attention to strong emotional responses from participants when accessing foods from FMs, such as feeling proud and empowered. By contrast, results from studies of food bank users’ identified a lack of nutritious, high-quality foods and limited food choices as significant concerns, leading to a loss of personal autonomy and dignity^(75,76)^.

The FMNCP also provided many opportunities to gain food and nutrition knowledge and skills. According to Freedman *et al.* ^(60)^, personal factors alone, such as food and nutrition knowledge, may be insufficient to improve food access. However, we found evidence that incorporating an education component within a multilevel intervention may have further enhanced food access and promoted healthy eating practices. Farmers and vendors played an important role in educating FMNCP participants about farming, various types of fruits and vegetables, and food preparation techniques. These types of interactions have been reported in qualitative studies of FM food subsidy programmes, suggesting that shopping in FMs may offer more opportunities for food and nutrition education than other food venues^(47,49)^. Moreover, participants with children reported that shopping in FMs increased their children’s exposure to fruits and vegetables and increased their opportunities to shop, prepare and cook foods with their children. Greater availability and exposure to fruits and vegetables may lead to increased consumption among children and youth^(77)^.

The FMNCP also addressed social factors related to food access as per Freedman *et al.*’s^(60)^ framework. Participants indicated that the FMNCP enhanced their social connections within and beyond FMs and yielded positive social and mental health outcomes. A few studies have noted that FM food subsidy programmes promoted positive social interactions^(36,48,49)^ and enhanced participants’ sense of community^(47)^. However, our study was unique in that we captured in-depth descriptions of participants’ social experiences and increased sense of community connectedness when shopping in FMs. The benefits of increased social connections for individuals who are experiencing food insecurity are significant. For instance, research in Québec, Canada, suggested that strong social cohesiveness within the household and the community can help prevent or reduce household food insecurity^(78)^. In addition, social supports promoted healthy eating practices amongst adults and older adults experiencing food insecurity^(79)^.

Our findings highlight key advantages of FM food subsidy programmes relative to the services offered by food banks. Food bank users have expressed the desire to socialise and connect with others around food and recommended food vouchers in grocery stores to purchase foods of their choice^(76,80)^. Moreover, perceptions of stigma, shame and embarrassment were common among food bank users, and feelings of ‘being fed’ without choice and queuing for food led to a loss of dignity^(76)^. By contrast, the FMNCP fostered autonomy, dignity and social connections among participants, which are essential in promoting greater participation, use and engagement with food assistance programmes in support of well-being and health^(81)^.

In addition to these positive experiences and outcomes, participants also reported constraints and challenges with the FMNCP. Participants living in rural communities experienced a lack of transportation and limited food availability and variety in FMs. Limited transportation is a commonly reported barrier to FM access^(47–51)^. In addition, Canadians living in rural and remote communities reported limited access to nutritious and affordable foods^(82)^. Based on an assessment of vendor performance in rural FMs, Schmit *et al.* ^(83)^ recommended four strategies to enhance access to nutritious foods in rural FMs: (1) strategic site selection and investments in market amenities, (2) changes in market policies and incentives to attract more vendors, (3) increased attention to the integration of FMs with other community events and (4) travel subsidies and coordinated regional and multi-community access to transportation.

Participants also experienced challenges when redeeming coupons in $3 increments, and the inability to access certain staple foods. Smaller coupon denominations or changing the paper coupon system to an electronic card-based system may increase purchasing flexibility and alleviate constraints with coupon increments. Participants in the Special Supplemental Nutrition Program for Women, Infants, and Children in the USA preferred the use of electronic cards to paper vouchers as it made purchasing foods more convenient and flexible^(84)^.

As the prevalence of food insecurity in Canada continues to rise^(1,85)^, there is a need for policies and interventions that increase access to nutritious, high-quality foods while prioritising autonomy, dignity and social inclusion^(76,81)^. Our findings have implications for FM food subsidy programmes, and the FMNCP in particular. We have therefore used study findings to inform recommendations to further enhance participants’ positive experiences and outcomes of FM subsidy programmes. First, as food inflation disproportionately impacts low-income households^(86)^, increasing food subsidies, particularly for larger households, will supplement participants’ food budgets, thereby freeing up funds that can be used for other living expenses. Increasing the amount of food subsidies for larger households may provide greater financial support and increased access to healthy foods for all household members. Second, expanding the programme to accept more participants may reduce waitlists and support more low-income households. Third, extending subsidies beyond 16 weeks or collaborating with other food subsidy or income support programmes to provide financial relief throughout the year may provide sustainable, long-term support for low-income households. For instance, the Supplemental Nutrition Assistance Program in the USA provides food subsidies to purchase foods in a variety of food venues, including FMs and grocery stores throughout the year^(87)^. This form of consistent and sustained income support, along with provincial and federal policy interventions such as social assistance, child benefits and employment insurance, is essential to tackle household food insecurity in Canada and elsewhere^(1)^.

The qualitative nature of the current study was a key strength as it allowed us to explore participants’ experiences and perceived outcomes of a FM food subsidy programme in an in-depth manner. Moreover, the use of Freedman *et al.*’s^(60)^ framework supported a theoretically informed understanding of factors that shaped food access for FMNCP participants. Nevertheless, some limitations should be noted as most of our participants were from rural communities (89 %). Therefore, our findings are most pertinent to the experiences and perceived programme outcomes of these groups. It is also possible that participants who agreed to be interviewed engaged more with the FMNCP and had more positive experiences with the programme than other participants. Finally, due to the FMNCP’s time-limited nature, our results underscore the need for future, in-depth studies of the longer-term impacts of these programmes with sustained participation.

## Conclusion

Participation in a FM food subsidy programme facilitated access to nutritious foods for low-income households and enhanced aspects of participants’ diet quality, well-being and health. The FMNCP provided financial support, increased access to fresh, high-quality fruits, vegetables and protein-rich foods and provided opportunities to develop food and nutrition knowledge and skills. Many participants also experienced increased social and community connections and perceived improvements in their social and mental health. On the other hand, environmental and programmatic constraints may have reduced programme participation and limited participants’ access to healthy foods in rural communities. Strategies such as increasing weekly subsidies, expanding the FMNCP to reduce waitlists and reach more low-income households and extending subsidies beyond the summer may help improve participants’ experiences and outcomes of FM food subsidy programmes.
